# Severe Suspected Anaphylaxis due to Sugammadex in a Healthy Patient Undergoing Endoscopic Sinus Surgery: A Case Report

**DOI:** 10.1155/cria/4537025

**Published:** 2026-04-21

**Authors:** Thai Quoc Phan, Quan Quang Luu, Thanh Minh Vo, Khuong Kinh Luu

**Affiliations:** ^1^ Department of Anesthesiology, Tam Anh General Hospital, Ho Chi Minh City, Vietnam

**Keywords:** anaphylaxis, erythema, general anesthesia, hypotension, rocuronium, sugammadex

## Abstract

**Background:**

Sugammadex is widely used for reversal of steroidal neuromuscular blockade and is generally considered safe; however, rare cases of anaphylaxis have been reported.

**Case Presentation:**

A 36‐year‐old healthy Vietnamese male patient underwent elective endoscopic sinus surgery under general anesthesia. Neuromuscular blockade induced by rocuronium was reversed with sugammadex (4 mg/kg). Within 5‐6 min after administration, shortly after arrival in the postanesthesia care unit, the patient developed acute dyspnea, generalized erythema and urticaria, severe hypotension, tachycardia, and bronchospasm, consistent with anaphylactic shock. Immediate treatment with epinephrine, fluid resuscitation, and airway support led to rapid stabilization. The patient recovered fully and was discharged on postoperative Day 3.

**Conclusions:**

Sugammadex‐induced anaphylaxis, although rare, can be life‐threatening even in individuals without prior allergy history. Vigilant monitoring, early recognition, and immediate epinephrine administration are essential to improve outcomes.

## 1. Introduction

Sugammadex is widely used in anesthetic practice to rapidly and reliably reverse neuromuscular blockade following administration of rocuronium or vecuronium and is generally considered safe. Its favorable profile, particularly the absence of muscarinic adverse effects seen with acetylcholinesterase inhibitors, has supported its routine use in modern anesthesia. Despite its overall safety, hypersensitivity and anaphylactic reactions to sugammadex have been increasingly recognized. The reported incidence of sugammadex‐induced anaphylaxis ranges from 0.02% to 0.04%, with responses typically occurring within minutes after intravenous administration, often during emergence from anesthesia or in the postanesthesia care unit (PACU) [1]. Notably, these events may arise even in patients without a prior history of allergy [2]. Although the underlying mechanism remains incompletely understood, immunoglobulin E‐mediated reactions to sugammadex or the sugammadex–rocuronium complex have been proposed [3–5].

We report a case of severe anaphylactic shock following sugammadex administration in a previously healthy adult, highlighting the importance of vigilance and prompt management of this rare but potentially life‐threatening complication.

## 2. Case Presentation

**FIGURE 1:**
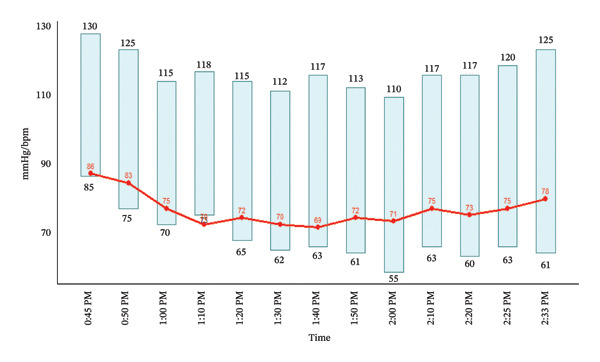
Intraoperative hemodynamic monitoring. Vertical bars indicate systolic (upper value) and diastolic (lower value) arterial blood pressure (mmHg), and the red line indicates heart rate (beats per minute). Measurements were recorded at regular intervals throughout the surgical procedure, demonstrating hemodynamic stability during anesthesia. Numeric labels denote the corresponding values at each time point.

A 36‐year‐old Vietnamese male patient (height 166 cm and weight 62 kg; body mass index 22.61 kg/m^2^) with no known medical comorbidities and no history of drug or food allergies was scheduled for elective endoscopic sinus surgery. The patient had previously undergone septoplasty under general anesthesia 2 years ago, during which 50 mg of rocuronium was administered. At the end of the procedure, neuromuscular blockade was reversed with 2.5 mg of neostigmine and 0.75 mg of atropine, with no recorded abnormalities or complications. In the current procedure, preoperative antibiotic prophylaxis was provided with ampicillin–sulbactam (1000/500 mg). General anesthesia was induced with 20 mcg of sufentanil, 140 mcg of propofol, and 40 mg of rocuronium (Rocuronium BFS, CPC1 Hanoi Pharmaceutical Company, Vietnam). Anesthesia was subsequently maintained with 2.5% sevoflurane in a mixture of oxygen and air throughout the procedure. The total duration of anesthesia and surgery was 70 min, during which the patient remained hemodynamically stable (Figure 1). Toward the end of surgery, analgesia was provided with paracetamol 1 g and nefopam 20 mg, and antiemetic prophylaxis with ondansetron 4 mg.

Neuromuscular blockade was reversed with sugammadex at a dose of 4 mg/kg. Approximately 3 min later, when the train‐of‐four ratio exceeded 0.9, the trachea was extubated. The patient was awake, breathing spontaneously, and hemodynamically stable on arrival in the PACU. Approximately 5‐6 min after sugammadex administration, the patient developed acute dyspnea, generalized erythema, and urticarial lesions involving the arms, chest, and abdomen. This was followed by rapid‐onset hypotension, tachycardia with weak peripheral pulses, and biphasic stridor, consistent with severe anaphylactic shock. The patient was immediately managed for suspected anaphylaxis with 0.5 mg of intramuscular (IM) epinephrine administered into the anterolateral thigh, followed by a continuous intravenous infusion at 0.1 mcg/kg/min via an 18‐gauge peripheral line. Continuous hemodynamic monitoring was established through a radial arterial catheter. To address distributive shock, fluid resuscitation was optimized using a passive leg raise test to guide the rapid administration of 0.9% normal saline at 20 mL/kg. Adjunctive pharmacological therapy included intravenous methylprednisolone (1 mg/kg) and diphenhydramine (1 mg/kg). Respiratory support was provided via bag‐mask ventilation with 100% oxygen, with preparations made for emergency tracheal reintubation if required.

**FIGURE 2:**
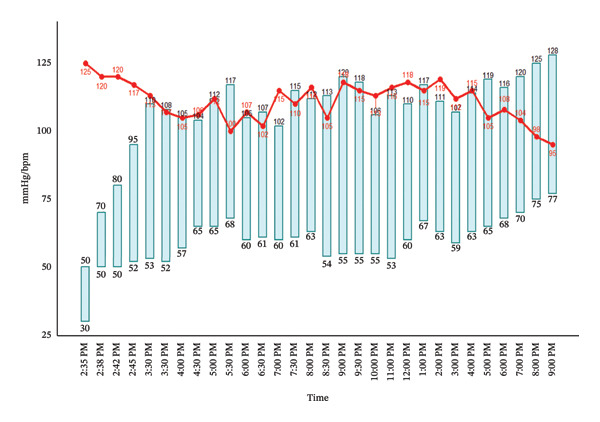
Postoperative hemodynamic monitoring in the postanesthesia care unit (PACU). Vertical bars indicate systolic (upper value) and diastolic (lower value) arterial blood pressure (mmHg), and the red line indicates heart rate (beats per minute). Measurements were recorded serially from admission to the PACU until transfer to the ward. Numeric labels denote the corresponding values at each time point.

After approximately 15 min of resuscitation, the patient’s hemodynamic status gradually stabilized (Figure 2). He remained conscious, breathing spontaneously with a patent airway, and peripheral perfusion improved. The epinephrine infusion was continued at 0.1 mcg/kg/min and gradually tapered over the subsequent hours. No biphasic reaction occurred. The patient was transferred to the general ward after stabilization, discharged on postoperative Day 3, and remained asymptomatic without sequelae at 6‐month follow‐up. Allergen skin testing was deferred after specialist consultation due to the potential risk of inducing recurrent severe anaphylaxis and the patient’s expressed preference to avoid further diagnostic interventions.

## 3. Discussion

Sugammadex is a neuromuscular blockade reversal agent that has been increasingly used in anesthetic practice owing to its rapid and predictable reversal of neuromuscular blockade, particularly for steroidal neuromuscular blocking agents such as rocuronium and vecuronium. Although sugammadex‐induced anaphylaxis is rare, its occurrence at critical time points, such as at the end of surgery, during patient transfer to or stay in the PACU, can make early recognition challenging and may pose a serious threat to patient safety.

To date, no cases of sugammadex‐related anaphylaxis have been reported in Vietnam. However, internationally, anaphylaxis associated with sugammadex has been documented in multiple centers, with reported incidence rates ranging from 0.02% to 0.04% [1]. A retrospective study in Japan involving 15,479 patients who received sugammadex identified only six cases of anaphylaxis, corresponding to an incidence of 0.039% [6]. In contrast, the incidence reported in Korea appears to be even lower, estimated at approximately 0.0014%–0.0143% [7]. Since the South Korean data were based on the number of sugammadex vials sold relative to reported anaphylaxis cases, the incidence rate in that study may be underestimated. Currently, limited evidence exists concerning the correlation between population demographics and the risk of sugammadex‐induced anaphylaxis.

In the present case, the patient developed severe anaphylactic symptoms shortly after arrival in the PACU, approximately 5‐6 min following administration of the neuromuscular blockade reversal agent. This finding is consistent with previous reports indicating that anaphylaxis related to sugammadex typically occurs very early, either immediately after intravenous injection or within a few minutes thereafter. According to Arslan et al., anaphylactic manifestations occurred within the first 5 min after sugammadex administration in up to 92.3% of patients [2]. Although the exact mechanism of sugammadex‐induced anaphylaxis remains incompletely understood, available evidence suggests that the reaction may be IgE‐mediated through the activation of mast cells and basophils [3]. Patients may exhibit hypersensitivity to sugammadex itself, to the complex formed between sugammadex and rocuronium, or potentially to both agents [5, 8]. In this report, the patient had a history of prior sinonasal surgery under general anesthesia using similar anesthetic agents, without any recorded allergic reactions. The key clinical difference was that in the patient’s previous anesthetic procedure, rocuronium‐induced neuromuscular blockade was reversed with neostigmine instead of sugammadex. The abrupt onset of characteristic symptoms, including generalized erythema, urticaria, hypotension, tachycardia, and bronchospasm immediately following sugammadex administration, was sufficient to establish a clinical diagnosis of severe anaphylaxis, fulfilling World Allergy Organization (WAO) criteria [9]. In the present case, rocuronium remains a potential causative agent. The previous anesthetic exposure may have facilitated IgE‐mediated sensitization, leading to the severe anaphylactic reaction observed during this second encounter. Rocuronium, a neuromuscular blocking agent containing quaternary ammonium epitopes that are widely distributed in environmental compounds, may cause hypersensitivity even at the first documented clinical exposure. Consequently, anaphylactic reactions to rocuronium most commonly occur shortly after anesthetic induction [10]. Following the administration of sugammadex, further progression of rocuronium‐induced hypersensitivity is considered unlikely, as sugammadex forms a tight inclusion complex with free rocuronium molecules, thereby reducing their bioavailability. Emerging evidence also suggests that sugammadex may attenuate or potentially reverse severe anaphylactic reactions specifically triggered by rocuronium [11, 12]. Therefore, the patient described in our case report could have developed an anaphylactic reaction to sugammadex alone or to the sugammadex–rocuronium complex.

Laboratory investigations such as serum tryptase, total IgE, and plasma histamine levels may support the diagnosis of an allergic reaction; however, identification of the specific allergen related to sugammadex generally requires intradermal or skin prick testing using diluted drug concentrations, which should be performed no earlier than 4 weeks after the anaphylactic event. Extreme caution is warranted when performing allergen identification tests in patients with a history of severe anaphylaxis. Yamaoka et al. reported a case of profound hypotension requiring epinephrine resuscitation and aggressive fluid therapy during intradermal skin testing with sugammadex in a patient who had previously experienced severe sugammadex‐induced anaphylaxis. Similar cases of severe anaphylaxis during intradermal testing have also been reported in patients with prior cefazolin‐induced anaphylaxis [13–15]. Following multidisciplinary consultation with clinical immunology specialists, allergen testing was not pursued because the patient declined further diagnostic evaluation, and there was a significant concern regarding the risk of recurrent severe anaphylaxis during testing.

Anaphylaxis, particularly in the operating room setting, often progresses rapidly and can be life‐threatening, constituting a medical emergency that requires prompt recognition and immediate treatment. Epinephrine is the first‐line therapy recommended for anaphylaxis, with rapid reversal of allergic manifestations, and its administration should not be delayed [9, 16]. Upon recognition of anaphylaxis, we immediately administered IM epinephrine into the anterolateral thigh, followed by a continuous intravenous epinephrine infusion. This was accompanied by airway and respiratory support, crystalloid fluid resuscitation to compensate for intravascular volume loss due to increased capillary permeability, and adjunctive therapies including antihistamines and corticosteroids, which reduce cytokine and prostaglandin synthesis through stabilization of mast cells and basophils. The patient was subsequently monitored in a fully equipped critical care setting to prevent biphasic anaphylactic reactions, which may occur within the first 6–12 h [17]. Early recognition and prompt treatment with epinephrine resulted in rapid clinical improvement, with significant resolution of severe anaphylactic symptoms within approximately 15 min of resuscitation. Hemodynamic stability was gradually restored, the patient remained awake and breathing spontaneously, and no signs of airway obstruction or organ hypoperfusion‐related complications were observed. Although its clinical application remains suboptimal, IM epinephrine continues to be recognized as the first‐line treatment for anaphylaxis. For healthcare providers, the recommended IM dosage is 0.01 mg/kg (up to a maximum of 0.5 mg), which can be simplified according to the guidelines of WAO. In contrast to the IM route, bolus administration of epinephrine carries a significant risk of potentially lethal cardiac arrhythmias. Consequently, intravenous delivery is not advised for the primary management of anaphylaxis. When clinical circumstances necessitate its use, it should be reserved for monitored settings and managed by clinicians proficient in the precise dilution and titration of the drug, preferably administered via a continuous infusion pump [9, 18].

Sugammadex‐induced anaphylactic shock remains a rare event, and current methods for allergen identification lack standardized dosing and concentration protocols. A limitation of this report is the lack of serum tryptase measurement due to the unavailability of reagents, as well as the absence of definitive allergen confirmation due to the inability to perform skin testing for patient safety reasons. Nevertheless, the distinct difference between the present reaction following sugammadex administration and previous anesthetic exposures, along with the timing of symptom onset and the classic clinical features of severe anaphylaxis, strongly supports the likelihood that sugammadex itself or the sugammadex–rocuronium complex was the causative agent. Written informed consent was obtained from the patient for publication of this case report.

## 4. Conclusions

Sugammadex‐induced anaphylaxis is a rare but potentially severe and life‐threatening complication that may occur even in patients without a prior history of allergy. The reaction typically develops rapidly after drug administration, particularly in the early postoperative period. Close clinical monitoring for at least 10–15 min after reversal of neuromuscular blockade with sugammadex, together with immediate administration of epinephrine at the first signs of anaphylaxis, is crucial to ensure patient safety and optimize outcomes.

## Author Contributions

T.M.V. was involved in the perioperative anesthetic management of this patient. T.M.V. and T.Q.P. collected the clinical data and drafted the manuscript as major contributors. Q.Q.L., T.Q.P., and K.K.L. critically reviewed and revised the manuscript.

## Funding

No financial support was provided for this study.

## Disclosure

All authors read and approved the final manuscript.

## Consent

Informed consent was obtained from the patient before manuscript submission.

## Conflicts of Interest

The authors declare no conflicts of interest.

## Data Availability

The data that support the findings of this study are available from the corresponding author upon reasonable request.
